# Dynamic Changes in Upper-Limb Corticospinal Excitability during a ‘Pro-/Anti-saccade’ Double-Choice Task

**DOI:** 10.3389/fnhum.2017.00624

**Published:** 2017-12-18

**Authors:** Luca Falciati, Claudio Maioli

**Affiliations:** Dipartimento di Scienze Cliniche e Sperimentali, Università degli Studi di Brescia, Brescia, Italy

**Keywords:** visually guided saccades, eye-hand coupling, transcranial magnetic stimulation, motor set, doublechoice task, antisaccade, motor evoked potentials

## Abstract

Under natural behavioral conditions, visually guided eye movements are linked to direction-specific modulations of cortico-spinal system (CSS) excitability in upper-limb muscles, even in absence of a manual response. These excitability changes have been shown to be compatible with a covert motor program encoding a manual movement toward the same target of the eyes. The aim of this study is to investigate whether this implicit oculo-manual coupling is enforced following every saccade execution or it depends on the behavioral context. Twenty-two healthy young adults participated in the study. Single-pulse transcranial magnetic stimulation was applied to the motor cortex at nine different time epochs during a double-choice eye task, in which the decision to execute a prosaccade or an antisaccade was made on the color of a peripheral visual cue. By analyzing the amplitude of the motor evoked potentials (MEP) in three distal muscles of the resting upper-limb, a facilitation peak of CSS excitability was found in two of them at 120 ms before the eyes begin to move. Furthermore, a long-lasting, generalized reduced corticomotor excitability develops following the eye response. Finally, a quite large modulation of MEP amplitude, depending on the direction of the saccade, is observed only in the first dorsal interosseous muscle, in a narrow time window at about 150 ms before the eye movement, irrespective of the type of the ocular response (pro-/anti-saccade). This change in CSS excitability is not tied up to the timing of the occurrence of the visual cue but, instead, appears to be tightly time-related to the saccade onset. Observed excitability changes differ in many respects from those previously reported with different behavioral paradigms. A main finding of our study is that the implicit coupling between eye and hand motor systems is contingent upon the particular motor set determined by the cognitive aspects of the performed oculomotor task. In particular, the direction-specific modulation in CSS excitability described in this study appears to be related to perceptual and decision-making processes rather than representing an implicit upper-limb motor program, coupled to the saccade execution.

## Introduction

In everyday life, we move the eyes in order to bring the image of a salient visual object on the fovea. Fast eye movements typically fall into two broad classes. One class stems from the bottom-up target selection triggered by a novel visual stimulus. Such ‘exogenously’ cued ocular responses can be assimilated to a visual grasp reflex (Hess et al., 1946) which involves little or even no cognitive control. By contrast, saccades can also voluntarily direct gaze toward a specific area of interest. The generation of such ‘endogenously’ cued saccades reflects additional processing requirements that involve, among others, goal-driven visual selection and top–down control of the oculomotor system.

Reflexive and volitional saccades are crucial for most common visually guided motor behaviors. Indeed, even if eyes and hands can move alone, a tight oculo-manual coordination is essential for an accurate execution of arm movements aimed at pointing, reaching or manipulating objects (Vercher et al., 1994; Henriques et al., 1998; Neggers and Bekkering, 1999; Horstmann and Hoffmann, 2005). Moreover, experimental evidence strongly supports the viewpoint that eye and arm motor systems are mutually coupled via extra-retinal signals and share, at least in part, a common neural controller (van Donkelaar and Staub, 2000; Engel and Soechting, 2003; Dean et al., 2011).

In this vein, recent transcranial magnetic stimulation (TMS) studies have reported phenomena of implicit coupling between eyes and hand during the execution of pure oculomotor tasks. Specifically, it has been shown that direction-specific changes of excitability in the upper-limb cortico-spinal system (CSS) occur following both reflexive (Falciati et al., 2013) and volitional saccades (Maioli and Falciati, 2015) toward a peripheral target, even though the task does not require any manual response and the arm is maintained at rest. Moreover, CSS excitability changes result to be tightly time-locked to saccade execution (Maioli and Falciati, 2015). Altogether, these data strongly support the thesis that whenever the eyes move, either under reflexive or volitional control, a sub-threshold motor plan encoding an aiming movement of the hand toward the gaze target is also activated.

However, it should be pointed out that other experimental data indicate that the implicit coupling between eyes and hand may depend on the context in which an ocular task is executed. In a previous TMS study (Maioli et al., 2007), it has been shown that changes of upper-limb CSS excitability are highly affected by forearm posture during the execution of smooth pursuit eye movements. Also in this case, excitability changes are compatible with a sub-threshold motor plan encoding a manual tracking of the gaze target. Interestingly, this modulation occurs if the forearm is held in a pronated posture, which is normally associated with the hand tracking of a visual object, but it is absent with a supinated posture of the forearm.

The aim of this study is to investigate whether a covert upper-limb motor program is issued in a mandatory way, whenever a saccade is executed, or whether the implicit coupling between eye and hand motor systems is contingent upon a particular set determined by the cognitive aspects of the performed sensorimotor task. The antisaccade paradigm, in which a volitional saccade is made to the opposite direction of a visual cue, imposes a very unnatural behavioral condition, creating a spatial incongruence between sensory stimulus and eye motor response. Therefore, it represents in many ways an ideal unfamiliar task to test whether a covert motor program for the hand could be possibly inhibited or, alternatively, could be generated with different spatial properties from those of the gaze shift. By contrast, in the presence of a forced coupling between eye and hand motor systems, direction-specific changes in the upper-limb CSS excitability should be determined exclusively by the side to which the gaze shift is directed, irrespective of the behavioral context in which a saccade is executed. By using TMS, in this study we analyze the time course of CSS excitability in the distal upper-limb muscles, while subjects perform a purely oculomotor task, in which the color of a cue determines whether the eyes must move toward the displayed visual stimulus (prosaccade) or toward its uncued mirror spatial location (antisaccade). Results show that the implicit coupling between eye and hand motor systems does not present constant features, but it is contingent upon the particular motor set determined by the cognitive aspects of the performed oculomotor task.

## Materials and Methods

### Subjects

Twenty-two adult volunteers (13 males and 9 females, mean age: 21.1 years, range: 20–22) with no history of head trauma or neurological disease participated in the study. All of the subjects had normal or corrected-to-normal vision, were right handed (as measured by the Edinburgh handedness inventory) and naïve to the purpose of the experiment. This study was conducted in accordance with the recommendations of the local Ethics Committee (Comitato Etico dell’Azienda Sanitaria Locale di Brescia) and with the ethical guidelines set forth by the Declaration of Helsinki. Written informed consent was obtained from all participants.

### Experimental Protocol

**FIGURE 1:**
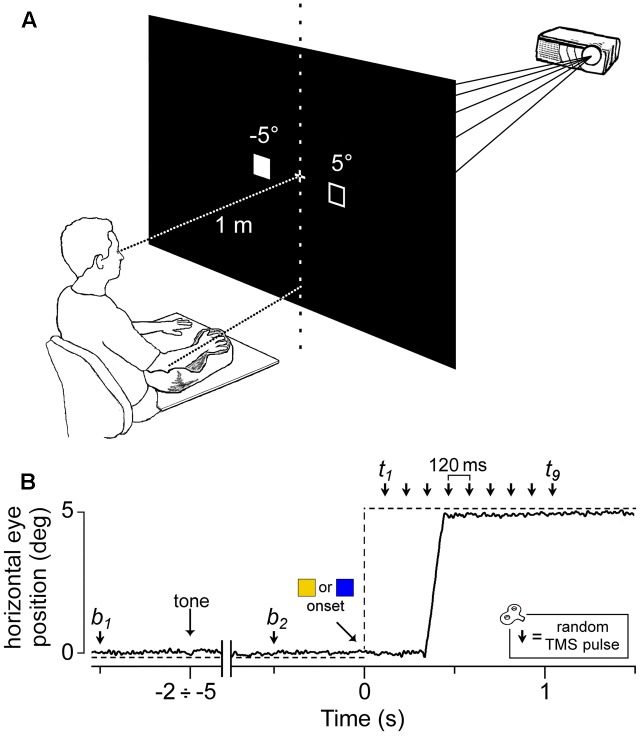
Experimental setup and protocol. **(A)** Visual stimuli were rear-projected on a wide-tangent black screen placed 1 m in front of the subject. The filled square represents the actual location of the visual cue, while the empty square indicates the other possible position. The head was immobilized using a chin rest and a head-support device (not shown). Notice the arm-hand posture imposed on the subject with respect to the central vertical meridian (see text). **(B)** An EOG recording (solid line) during a representative saccadic response. The dashed line indicates the time course of the visual stimulation. The peripheral cue (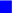 or 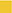) appeared at a variable time interval of 2–5 s after a warning tone. A single pulse of TMS was randomly delivered either before cue onset (at *b*_1_ or *b*_2_) or at one of nine equally spaced time delays between 120 ms and 1080 ms (*t*_1_–*t*_9_).

The subjects were sitting with their right upper-limb resting in a relaxed position on a horizontal support. The support consisted in a polystyrene bead vacuum splint and was molded to the hand, palm and forearm of the subject. This device enabled the limb to be loosely restrained to maintain the horizontal alignment of the longitudinal axis of the pronated hand with the forearm and to keep it pointing toward the central vertical meridian (**Figure 1A**). The head was stabilized using a combination chin rest and head support device. Visual stimuli were rear projected on a wide-tangent black screen (160 cm in width and 120 cm in height) placed 1 m in front of the subject. Participants were instructed to fixate a white central cross, which remained on throughout the duration of a given trial. After a variable time interval of 2–5 s after a warning tone, a colored square (subtending 0.6° of visual angle) appeared for 2 s at 5° from the central cross along the horizontal meridian, in the left or right visual field. The color of the peripheral stimulus was randomly set to be blue or yellow and represented the imperative cue that signaled the subjects to quickly respond by moving their gaze either to the square (‘prosaccade’ condition), or, conversely, to its mirror image location on the horizontal meridian (‘antisaccade’ condition). Color code for making a pro- or an anti-saccade were also varied at random among participants. Subjects were told to make an eye movement toward the peripheral visual stimulus or toward its mirror spatial position according to the stimulus color code and to maintain their gaze on the attained location until the turning off of the imperative stimulus, which marked the beginning of a new trial. Participants were encouraged to make the correct oculomotor response on the basis of the color of the visual cue.

Within each trial, a single-pulse TMS was delivered on the left motor cortex to record motor evoked potentials (MEPs) from three relaxed muscles of the contralateral upper-limb (*first dorsal interosseous*, FDI; *abductor digiti minimi*, ADM; *extensor carpi radialis*, ECR). The TMS pulse randomly occurred at one of 11 possible time epochs (**Figure 1B**): 500 ms before the warning tone (*b*_1_), 500 ms before the onset of the peripheral cue (*b*_2_) or at one of 9 different epochs between 120 ms to 1080 ms after it, corresponding to multiples of 120 ms time delays (*t*_1_ to *t*_9_). At *b*_1_ and *b*_2_, TMS was delivered before cue occurrence to measure the excitability of recorded muscles in the resting condition (‘baseline stimuli’).

Ocular response (pro-/anti-saccade), gaze direction (leftward/rightward) and TMS time epochs (‘baseline’/‘test’ stimuli) were randomly intermixed within the experimental session, so the subjects were unable to predict the eye task to perform and the timing of the TMS occurrence. Each experimental session comprised 5 blocks of 76 trials (with 3 min intervals between blocks), yielding an overall number of 380 trials [10 trials for each of the 2 baseline stimuli plus 10 trials for each of the 36 possible test conditions (2 sides × 2 ‘prosaccade’/‘antisaccade’ conditions x 9 time delays)]. The assignment of ‘prosaccade’/‘antisaccade’ condition to the color of the peripheral imperative stimulus was randomly balanced across subjects.

Particular attention was paid when explaining the task to avoid drawing the subject’s attention to the possibility of making an aiming movement of the arm toward the target. In addition, the lack of any imagery of manual pointing movements was assessed through a subject interview after the experimental session. Accuracy in task performance was defined as the percentage of trials in which the subject made the correct type of oculomotor response (pro-/anti-saccade), according to the color of the visual cue.

### Eye Movement and EMG Recording

Horizontal and vertical eye movements were recorded (DC 200 Hz low-pass filtered) by means of electrooculography (EOG). Ag-AgCl electrodes were placed at the external canthi of both eyes and above and below the right eye. During an experimental session, EOG calibration was repeated at each interval between trial blocks. Drift of DC offset was compensated within each trial by making the subject look at the central fixation cross before task onset. Surface electromyograms (EMG) were simultaneously recorded on the right-hand side from FDI, ADM and ECR muscles (1000× amplification; 0.2 Hz – 2 kHz bandwidth). Besides being easily stimulated by low intensity TMS, these muscles were selected because they exhibit direction-specific excitability changes following visually guided saccades (Falciati et al., 2013; Maioli and Falciati, 2015). Attention was paid to ensure that the subjects kept their muscles completely relaxed, as demonstrated by the absence of any detectable EMG activity for the entire duration of the task.

The EOG and EMG signals were digitally converted at a sampling rate of 5 kHz (National Instruments PCI-MIO-16E-4) and analyzed offline by means of custom LabVIEW (National Instruments, Austin, TX, United States) software. The onset of the saccadic response was evaluated by visual inspection of each individual trial. Saccade latency was measured by the analysis software by identifying the peak eye acceleration, occurring within a manually indicated time epoch around the beginning of the ocular response.

### Transcranial Magnetic Stimulation

Transcranial magnetic stimulation procedures have been explained in detail in a previous study (Maioli and Falciati, 2015). Single pulse, biphasic waveform stimuli were delivered by a 70 mm figure-eight double coil connected to a MagStim Super Rapid magnetic stimulator (Mag-1450-00, MagStim Co. Ltd., Whitland, United Kingdom) that was positioned over the left motor cortex. The coil was placed tangentially to the scalp, with the handle pointing backward and laterally at a 45° angle to the sagittal plane. The scalp site at which MEPs were elicited in the FDI muscle at the lowest stimulus strength was determined. Once the optimal scalp site was found, the coil was securely fixed in place by means of an appropriate mechanical device. The response threshold was defined as the stimulus intensity at which 5 out of 10 consecutive single TMS pulses evoked MEPs with an amplitude of at least 100 μV in the relaxed muscle. At the optimal scalp site for FDI, an appropriate stimulation intensity also evokes MEPs in the ADM and ECR muscles, although usually of smaller amplitude. Therefore, during the entire stimulation paradigm, stimulus intensity was set at 1.2 times the FDI motor threshold, to ensure that a reliable response was elicited also in the other two muscles. The mean stimulation intensity across subjects was equal to 68.4% (range: 48–78%) of the maximum power of the magnetic stimulator.

### Data Processing

Single MEP outliers for each TMS delay were identified by the Grubbs’ test with *p* < 0.01 and were removed from the entire data set. Furthermore, to ensure that the excitability changes were measured against a reliable baseline, subjects were included in the analysis only if, within each muscle, the MEPs obtained with *b*_1_ and *b*_2_ stimuli had a mean amplitude greater than 50 μV. This acceptance criterion was fulfilled in 22, 21 and 20 subjects for FDI, ADM and ECR muscles respectively.

In order for a trial to be included in the analysis, the following criteria had to be fulfilled: (1) upper-limb muscles were maintained completely relaxed, as defined by the absence of any detectable EMG activity during the entire trial duration; (2) the ocular behavior (pro-/anti-saccade) was coherent with the response demanded by the color code of the peripheral imperative stimulus. For each muscle, the detection of a spontaneous contraction during the task yielded an average exclusion of 4.0% of the trials (range: 0.0–13.3%).

The peak-to-peak amplitudes of the MEPs recorded from the investigated muscles were measured trial by trial. MEP amplitudes were normalized within each subject by a baseline measure computed for each muscle by averaging *b*_1_ and *b*_2_ responses, i.e., during fixation of the central cross prior to the warning tone and the visual stimulus, respectively. In fact, a *t*-test applied separately for the FDI, ADM and ECR muscles within each subject, to compare the mean MEP amplitudes obtained at the TMS delays *b*_1_ and *b*_2_, never yielded a statistically significant difference (*p* > 0.06 in all comparisons).

Mean values across subjects (and mean standard deviation within subjects, SDw) for *b*_1_ and *b*_2_ MEP amplitudes were 1.237 mV (SDw = 0.935 mV) and 1.304 mV (SDw = 0.872 mV) for FDI, 0.671 mV (SDw = 0.525 mV) and 0.719 mV (SDw = 0.452 mV) for ADM, 0.226 mV (SDw = 0.157 mV) and 0.261 mV (SDw = 0.153 mV) for ECR, respectively.

In order to ascertain whether the changes in muscle excitability were time-locked to saccade execution instead of being elicited by the presentation of the visual stimulus, CSS excitability was also investigated by analyzing MEP amplitudes after TMS delays were recomputed with respect to the beginning of the ocular response, identified on the basis of the peak of eye acceleration. In the rest of the paper, we shall refer to this data analysis as to *saccade-locked*, as opposed to *stimulus-locked* analysis, in which TMS delay was measured with respect to the onset of the visual stimulus.

### Statistical Analysis

The normalized MEP amplitudes were analyzed using linear mixed-effects regression, LMER, with the lme4 package (Bates et al., 2015) in the R environment (R Core Team, 2016). We obtained *p* values for regression coefficients using the car package (Fox and Weisberg, 2011). In contrast to a more traditional approach with data aggregation and repeated-measures ANOVA analysis, LMER allows controlling for the variance associated with random factors without data aggregation (see Baayen et al., 2008; Judd et al., 2012). This approach is particularly suitable for the analysis of *saccade-locked* data as, contrary to *stimulus-locked* data, the independent variable ‘TMS time delay’ can assume any value, without any obvious aggregation determined by the experimental design. In order to apply a homogeneous statistical analysis, we applied a linear mixed-effect regression to both data sets.

For the regression model, ‘*response*’ (prosaccade vs. antisaccade) and ‘*gaze direction*’ (leftward vs. rightward) were tested as fixed-effect categorical predictors of the changes in MEP amplitude, on top of the background changes in CSS excitability, represented by the effects bound by the continuous variable ‘*time.*’ Furthermore, in order to take into account a possible time-dependent effect of the two categorical variables, the linear interactions between ‘*time*’ with both ‘*response*’ and ‘*gaze direction*’ were also added to the model. The time effect on the normalized MEP amplitude was taken as random at the participant level (grouping factor), to control for the influence of the different inter-subject modulation in background CSS excitability during the experimental trial. As explained in the Results section, time has been introduced in the regression model as a cubic polynomial both as fixed and as random effect, to obtain a better fitting of the experimental data. Preliminary analysis has shown that introducing a cubic polynomial in the model leads to a significant drop in deviance with respect to a quadratic and linear time effect, as assessed by a χ^2^ statistics, testing the difference in deviance between successive models. For the sake of brevity, in the following we shall present only the χ^2^ tests from the LMER results (type II Wald χ^2^ tests), to evaluate the significance of the regression coefficients.

## Results

### Saccadic Latencies

The double-choice task yielded quite long response latencies, with a large statistical variability within each subject. This variability was exploited to provide a saccade-linked description of CSS excitability modulation, by computing mean MEP amplitude at binned time intervals with respect to saccade onset (see below).

Mean (±SD) latencies across participants were 385 ± 59 ms (median = 377 ms) for ‘prosaccade’ trials and 433 ± 73 ms (median = 424 ms) for ‘antisaccade’ trials. The average latencies of the two response conditions differed in a statistically significant manner [two-tailed paired *t*-test: *t*(21) = 7.029, *p* < 0.001]. All subjects performed the task with high accuracy, as only 6.6% of the trials were rejected on average for errors in the type of the executed oculomotor response (pro-/anti-saccade), as settled by the color of the visual cue (intra-subject range: 0.0–15.4%).

### Changes in the Overall CSS Excitability

**FIGURE 2:**
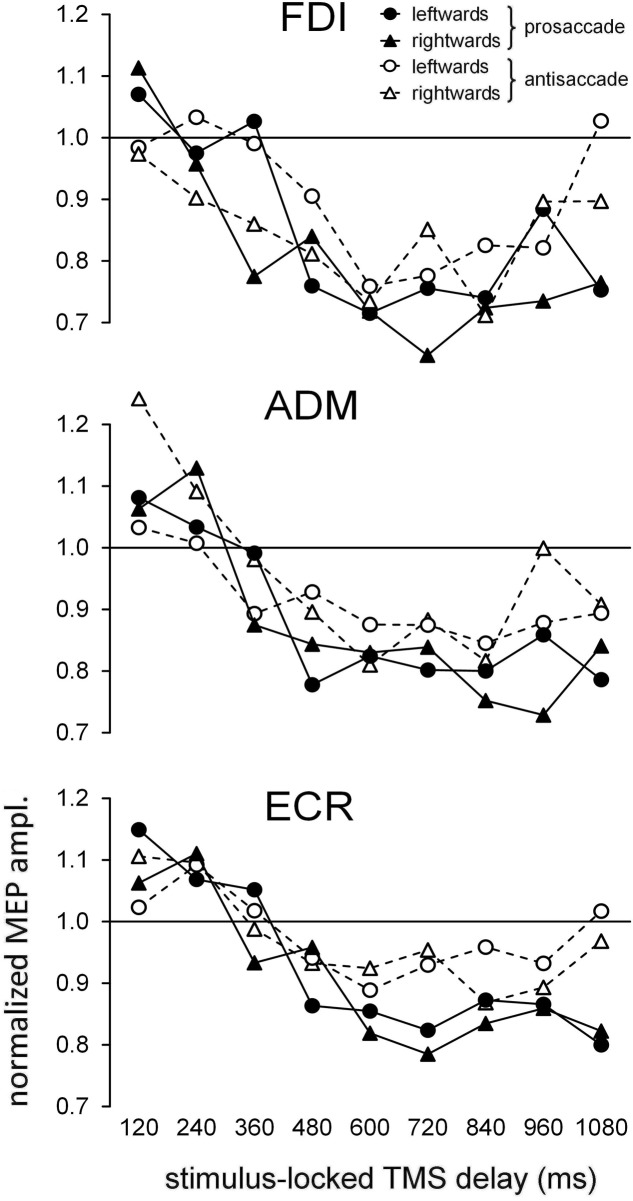
Time course of the changes in the mean normalized motor evoked potential (MEP) amplitudes as a function of ocular task (‘prosaccade’/‘antisaccade’) and gaze direction (‘leftward’/‘rightward’). Graphs depict, separately for each recorded muscle, the data values as a function of the TMS delay with respect to the onset of the peripheral visual stimulus. Values higher than unity along the *Y*-axis indicate MEP amplitudes larger than baseline.

**Figure 2** depicts, for each recorded muscle, the mean MEP amplitudes (normalized to the baseline value within each subject) as a function of time epoch of TMS pulse, type of ocular response and direction of gaze shift. A simple inspection attests the occurrence of a substantial modulation in MEP amplitude depending on the TMS delay. Specifically, a marked decrease in CSS excitability below baseline is clearly present in all three recorded muscles, starting at about 400 ms after the onset of the visual stimulus.

**FIGURE 3:**
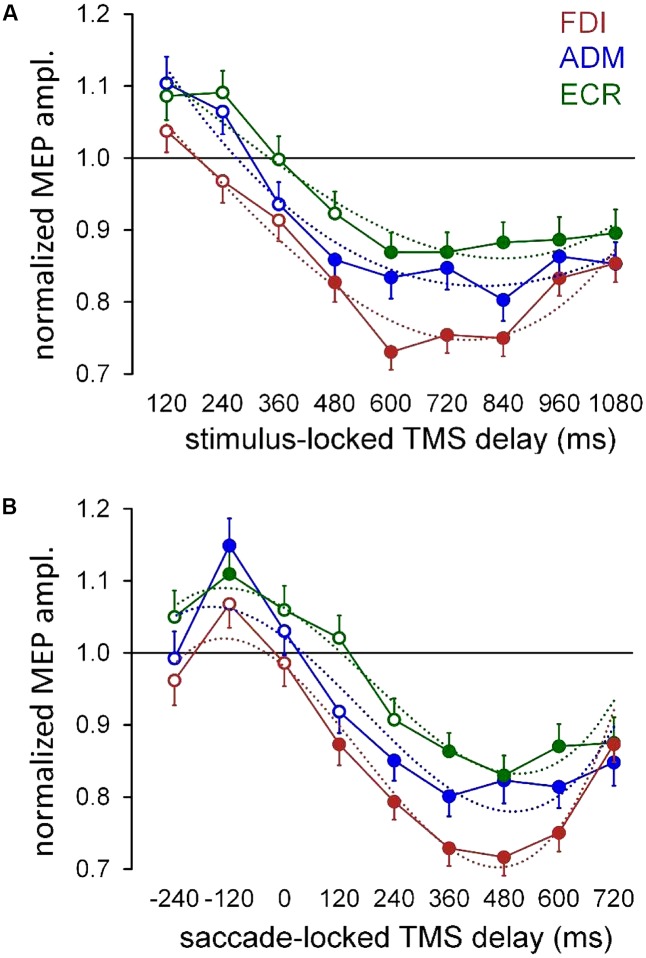
Mean MEP amplitudes across subjects irrespective of ocular task condition and gaze direction. **(A)** Mean values were computed for each muscle as a function of TMS delay with respect of the onset of the peripheral visual stimulus (‘stimulus-locked TMS delay’). **(B)** Mean values were obtained by grouping the TMS delays with respect to the beginning of the ocular response, in equally spaced bins of 120 ms (‘saccade-locked TMS delay’). Figures drawn along the *X*-axis represent the bin central values used for the averaging procedure. Filled symbols denote a statistically significant difference (*p* < 0.05) from the baseline (see text). Values higher than unity indicate MEP amplitudes larger than baseline. Error bars represent the standard error of the mean. In both graphs, thin dotted curves depict the least-squares best fitting of the experimental data with a cubic polynomial.

To provide a description of the time course of the modulation in the overall CSS excitability, the mean normalized MEP amplitude was computed for each TMS delay across all participants, irrespective of the ‘pro-/anti-saccade’ task and side of the oculomotor response (**Figure 3A**). Furthermore, in order to ascertain whether the timing of the excitability changes are more tightly correlated with the presentation of the visual stimulus or with the execution of the oculomotor response, we recomputed mean MEP amplitudes across all participants, after grouping TMS delays with respect to the saccade onset in equally spaced bins of 120 ms (**Figure 3B**). In both panels, filled symbols indicate that the mean MEP amplitude for that TMS delay falls outside the 95% one-side confidence interval testing equality with baseline measurements, by applying the Dunnett’s method for multiple comparisons (two-way ANOVA with ‘*time bin*’ and ‘*participant*’ as grouping factors).

**Figure 3** shows that changes in the overall CSS excitability are more tightly synchronized with the eye movement execution than with the presentation of the visual stimulus. In fact, in the graph with *saccade-locked* data (**Figure 3B**), a clear build-up of the normalized MEP amplitude in ADM and ECR muscles, occurs before the ocular response, reaching its peak at about 120 ms before saccade onset. It should also be noticed that, mean MEP amplitudes in these muscles do not differ from baseline at 240 ms before the eye movement execution, which corresponds, in the great majority of trials, to a time epoch well after the presentation of the visual cue. Furthermore, data clearly demonstrate that in all muscles excitability levels turn into a deep and long-lasting reduction after the execution of the eye response. Accordingly, in the graph showing *stimulus-locked* data (**Figure 3A**), the decrease of CSS excitability below baseline level seems to occur in all muscles after the oculomotor response, as suggested by the value of the median saccadic latency (397 ms), measured irrespectively of the execution of a pro- or anti-saccade. Finally, in the FDI muscle, the post-saccadic excitability depression appears more profound than that of ADM and ECR, and the MEP amplitude increase during the latency of the oculomotor response is absent.

In order to ascertain whether the type of eye response and the direction of the gaze movement have an effect on the time course of CSS excitability changes, a linear mixed-effects model was applied to the intra-subject normalized amplitudes of single MEPs within each muscle (see Materials and Methods). By this analysis, ‘*response’* (prosaccade vs. antisaccade) and ‘*gaze direction*’ (leftward vs. rightward) are tested as fixed-effect categorical predictors of the changes in MEP amplitude on top of a background time-variant modulation in CSS excitability during the task. In *stimulus-locked* data, the time variable is simply represented by the TMS delay from the visual stimulus. In *saccade-locked* data, the time delay is computed for each MEP as the interval between TMS and the saccade onset. After inspection of **Figure 3**, the time course of the background excitability modulation appears to be properly modeled by a cubic polynomial. A least-squares fitting of mean MEP amplitudes by a cubic polynomial, shown for each muscle by thin dotted curves in both *stimulus-locked* (**Figure 3A**) and *saccade-locked* panels (**Figure 3B**), attests to the adequacy of the model. Therefore, time has been introduced in the regression model as a cubic polynomial both as fixed and as random effect. Finally, in order to test a time-dependent effect of the two categorical variables, the linear interactions between ‘*time’* with both ‘*response’* and ‘*gaze direction*’ were also added to the model. For *saccade-locked* data, the statistical analysis was restricted to TMS delays comprised between 250 ms before and 700 ms after the beginning of the gaze response. **Tables 1**, **2** report the statistical significance of the fixed-effect regression coefficients estimated by the mixed-model regression fit on the *stimulus-locked* and *saccade-locked* data, respectively.

**Table 1:** Linear mixed-effects model fit of normalized MEP amplitudes on *stimulus-locked* data.

	FDI (N subj. = 22)		ADM (N subj. = 21)		ECR (N subj. = 20)	
	Wald χ^2^	*P*	Wald χ^2^	*P*	Wald χ^2^	*P*
Response	**6.962**	**0.008**	**9.077**	**0.003**	**11.251**	**0.001**
Gaze direction	**6.983**	**0.008**	0.505	0.477	0.967	0.326
Poly (time, 3)	**30.466**	**0.000**	**21.335**	**0.000**	**17.085**	**0.001**
Response × gaze direction	0.071	0.790	1.693	1.193	0.052	0.819
Response × time	**10.489**	**0.001**	1.802	1.179	**9.808**	**0.002**
Gaze direction × time	0.000	0.994	0.645	0.422	0.145	0.703
Response × gaze direction × time	0.169	0.681	0.482	0.488	2.127	0.145

*Fixed effects statistics. Bold figures mark statistical differences (*p* < 0.05)*.

**Table 2:** Linear mixed-effects model fit of normalized MEP amplitudes on *saccade-locked* data.

	FDI (N subj. = 22)		ADM (N subj. = 21)		ECR (N subj. = 20)	
	Wald χ^2^	*P*	Wald χ^2^	*P*	Wald χ^2^	*P*
Response	**5.215**	**0.022**	**6.771**	**0.009**	**10.132**	**0.001**
Gaze direction	**7.808**	**0.005**	0.436	0.509	1.217	0.270
Poly (time, 3)	**41.979**	**0.000**	**28.753**	**0.000**	**21.870**	**0.000**
Response × gaze direction	0.153	0.902	2.005	0.157	0.083	0.773
Response × time	**16.894**	**0.000**	**10.663**	**0.001**	**15.831**	**0.000**
Gaze direction × time	0.276	0.599	1.329	0.249	1.231	0.267
Response × gaze direction × time	0.099	0.753	0.069	0.793	1.800	0.180

*Fixed effects statistics. Bold figures mark statistical differences (*p* < 0.05)*.

As expected, the statistical analysis demonstrates a significant dependence of CSS excitability on the time cubic polynomial [‘*poly (time, 3)*’ in the tables] in all muscles. Interestingly, ‘*response*’ factor affects in a significant manner MEP amplitude in all muscles with both *stimulus-locked* and *saccade-locked* data. When TMS delay is computed with respect to saccade onset, a significant linear interaction with time is also present in all muscles. By contrast, in *stimulus-locked* data the interaction ‘*response* × *time*’ gives a statistically significant better fitting only in FDI and ECR muscles. Finally, a principal effect of ‘*gaze direction*’ is present only in the FDI muscle, both in *stimulus-locked* and in *saccade-locked* data.

### Effects of the Direction of the Ocular Response on CSS Excitability Changes

**FIGURE 4:**
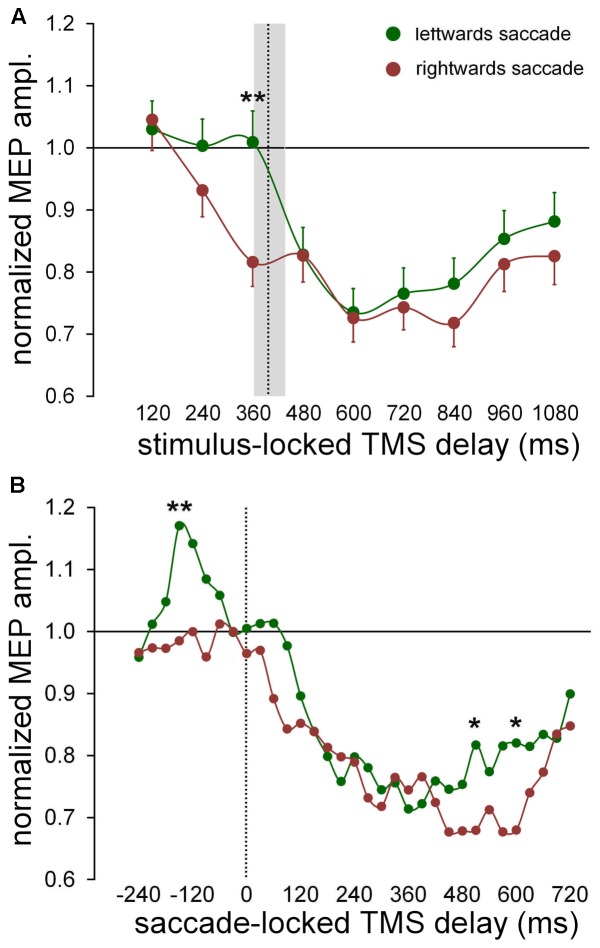
Changes of excitability in FDI muscle as a function of saccade direction. Data points were computed by averaging normalized MEP amplitudes across subjects irrespective of type of the oculomotor response (pro-/anti-saccade). Continuous lines represent a spline interpolation of the data. **(A)** Mean values were computed as a function of TMS delay with respect of the onset of the visual stimulus (‘stimulus-locked TMS delay’). The vertical dotted line depicts the median value of saccade latencies and the grey shade indicates the interquartile range. Error bars represent the standard errors of the mean. **(B)** Data points represent a moving average computed every 30 ms, on saccade-locked TMS delays falling within a range of ± 60 ms around the plotted value. Vertical dotted line marks the saccade onset. ^∗^ and ^∗∗^ denote a statistically significant difference between leftward and rightward saccades with *p* < 0.05 and *p* < 0.01 (type II Wald χ^2^ tests), respectively.

Statistical analysis demonstrates that a significant principal effect of ‘*gaze direction*’ on MEP amplitude occurs only in the FDI muscle, with no statistical interaction with the factor ‘*response.*’ That is, CSS excitability for this muscle is modulated by the side toward which the saccadic response is directed, irrespective of whether a prosaccade or antisaccade is executed. **Figure 4A** illustrates the changes of FDI excitability as a function of TMS delays with respect to the onset of the visual stimulus (*stimulus-locked*), by averaging normalized MEP amplitudes across participants for leftward or rightward saccades, independently of the type of oculomotor response. The graph shows that, accordingly with the results of the regression analysis, CSS excitability was reduced to a greater extent when the eye response was directed toward the right-hand than the left-hand. This difference between trials with leftward and rightward saccades is most noticeable at 360 ms after stimulus onset. By conducting a mixed-effect regression analysis on single MEP amplitudes for each TMS delay, on a model in which only ‘*gaze direction*’ was taken as both fixed and random effect at the participant level, we found that its fixed-effect regression coefficient was significant only at the TMS delay of 360 ms [type II Wald χ^2^(1) = 9.005; *p* = 0.003]. It should be noticed that this time epoch precedes the value of the median saccadic latency (397 ms), represented in **Figure 4A** by a vertical dotted line together with the inter-quartile range. Thus, the analysis of *stimulus-locked* data indicate that FDI CSS excitability is slightly higher for most of the task duration when the eye response is directed to the left-hand side, but this excitability difference becomes much larger shortly before the saccade execution.

We conducted the analysis of the *saccade-locked* data with the aim to investigate whether the excitability changes bound to the direction of the oculomotor response are more tightly time-correlated to the execution of the eye movement than to the occurrence of the visual stimulus. In **Figure 4B**, normalized MEP amplitudes are represented as a function of the TMS delay with respect to the saccade onset, by a moving average computed every 30 ms, performed on all TMS delays falling within a range of ±60 ms around the plotted value. Mean MEP amplitudes were computed separately for trials with leftward and rightward eye movements. This procedure allows to study excitability changes with a pretty good time-resolution and a sufficient level of low-pass filtering to smooth the experimental noise of the data. It can be clearly seen that the two curves diverge in a more pronounced manner around 150 ms before saccade onset and very late during the trial, roughly between 500 and 600 ms. A mixed-effect regression analysis of MEP amplitudes within each individual time bin, with ‘*gaze direction*’ as both fixed and random effect at the participant level, yielded that the fixed-effect regression coefficient was statistically significant only at the following TMS delays: -150 ms [χ^2^(1) = 6.807; *p* = 0.009], 510 ms [χ^2^(1) = 4.136; *p* = 0.042] and 600 ms [χ^2^(1) = 5.494; *p* = 0.019]. All other delays were not statistically significant (*p* > 0.06).

Analyzing more in detail the excitability modulation in the FDI muscle during the time interval embracing the ocular response, one can see that MEP amplitude does not change from baseline level up to the saccade onset, when the eye movement has to be made toward the ipsilateral side (with respect to the recorded muscle). By contrast, when the eye response is directed toward the contralateral side, CSS excitability abruptly increases by almost 20% within a narrow time epoch (lasting approximately 100 ms), with a peak value occurring at about 150 ms before the saccade onset. Around the beginning of the eye response, FDI excitability has come back to baseline, turning shortly after into the already described profound, long-lasting post-saccadic depression.

The significant difference in MEP amplitude between leftward and rightward saccade trials, found at 500–600 ms after the oculomotor response occurs at such a delayed latency, that it is very difficult to find a reliable interpretation for it. It may reflect a cortical excitability modulation associated to the intention of a voluntary action and it will not be discussed any further.

To summarize, the analysis of MEP amplitudes strongly suggests that the side-dependent modulation of CSS excitability in FDI muscle is more tightly synchronized to the onset of the oculomotor response, rather than to the timing of presentation of the visual stimulus. This short-lasting excitability modulation appears to be independent of the oculomotor task (pro-/anti-saccade), being just related to the direction of the gaze shift. Its occurrence within a narrow time window preceding the eye response, is highly indicative that this CSS excitability modulation is correlated to the decision-making process, based on the color discrimination of the visual cue, about the direction of the gaze shift to be made.

### Effects of Ocular Task on CSS Excitability Changes

LMER analysis demonstrated in all muscles a significant effect of the factor ‘*response,*’ with both *stimulus-locked* (**Table 1**) and *saccade-locked* data (**Table 2**). Furthermore, a statistically significant ‘*response* × *time*’ interaction was also found in all regression analyses, except in the ADM muscle when TMS delays are measured with respect to the timing of the visual stimulus. A significant interaction indicates that the change in CSS excitability that is determined by the execution of a pro- or an anti-saccade is not constant during the task, but varies with time.

**FIGURE 5:**
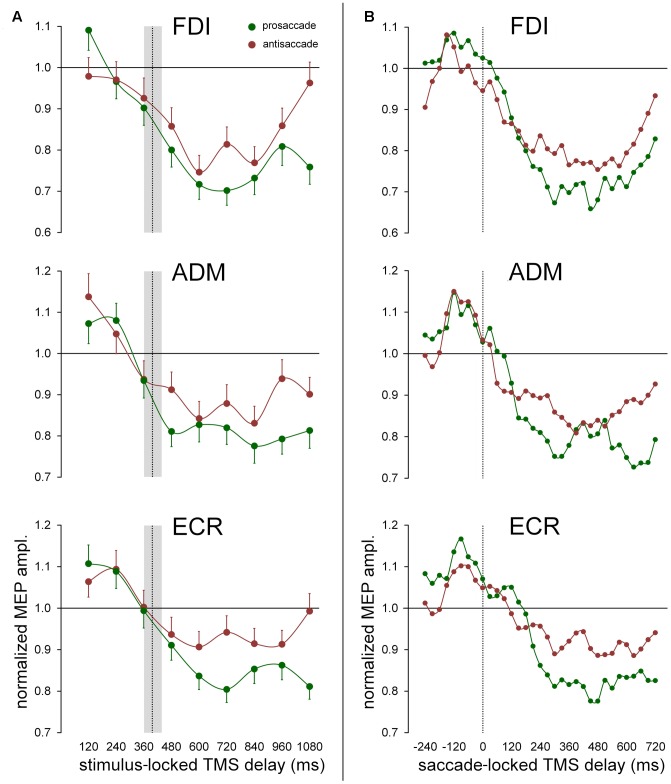
Effect of the ocular task on mean MEP amplitudes. Green and red data points represent mean values across subjects for pro- and anti-saccades respectively, irrespective of gaze direction. Continuous lines represent a spline interpolation of the data. **(A)** Mean values were computed as a function of TMS delay with respect of the onset of the visual stimulus (‘stimulus-locked TMS delay’). The vertical dotted line depicts the median value of saccade latencies and the gray shade indicates the interquartile range. Error bars represent the standard errors of the mean. **(B)** Data points represent a moving average computed every 30 ms, on saccade-locked TMS delays falling within a range of ±60 ms around the plotted value. Vertical dotted line marks the saccade onset.

The time-varying characteristics of the effect on MEP amplitude of the type of the ocular response (irrespective of eye movement direction) are graphically illustrated for all muscles in **Figure 5A** (*stimulus-locked* data) and in **Figure 5B** (*saccade-locked* data). Mean values of the normalized MEP amplitudes are computed with the same procedure described in Section “Effects of the Direction of the Ocular Response on CSS Excitability Changes,” for both *stimulus-locked* and *saccade locked* analysis of the TMS delays. Graph inspection neatly demonstrates that the statistically significant ‘*response × time’* interaction is determined by the deeper reduction in excitability that develops in the course of the prosaccade trials, with respect to the trials in which an antisaccade is performed. Interestingly, looking at the *saccade-locked* graphs, MEP amplitude begins to decrease in all muscles after the execution of the eye movement in a similar manner independently of the type of the saccadic response and direction (cfr. **Figure 4B** for FDI muscle; not shown for ADM and ECR). Only at about 200 ms after saccade onset, a deeper excitability decrease emerges in all muscles in prosaccade trials, lasting for all the remaining of the recording time in FDI and ECR. By contrast, this differential modulation in CSS excitability appears to have a biphasic time course in the ADM muscle, as the decrease in MEP amplitude becomes similar, in both pro- and anti-saccade trials, in the time interval from about 400 ms to 500 ms after the saccade onset. A precise timing of the differential behavior in CSS excitability between pro- and anti-saccade trials is more difficult to assess in *stimulus-locked* data. However, one can clearly notice that, in all muscles, the more pronounced decrease of MEP amplitude in the prosaccade trials begins on average after the execution of the oculomotor response (**Figure 5A**).

## Discussion

Subjects performed a double-choice task, in which the decision to execute volitional saccades toward either a peripheral visual stimulus (prosaccade) or an uncued spatial location (antisaccade) depended on the discrimination of the color of a visual cue. Mean saccade latencies in our study are somewhat longer than those commonly reported in other papers with a similar interleaved paradigm for pro- and anti-saccades (cfr. Zeligman and Zivotofsky, 2017; see however Irving et al., 2009). Furthermore, participants managed to execute the task with a very low error rate (6.6%). A possible reason for these differences may lay in the fact that we encouraged subjects to perform the task with high accuracy, in order to keep as large as possible the number of valid trials (in view of the large variability of MEP amplitude). Furthermore, dissimilarly from most previous studies, we adopted an overlap paradigm, in which the central fixation point was kept visible throughout the task. It is well known that this situation yields reaction times that are sensibly longer than when central fixation is turned off at the presentation of the visual cue. Interestingly, the mean latency of prosaccades in the present study is virtually identical to that reported in Maioli and Falciati (2015), where we applied a ‘go/no-go’ paradigm with a very similar experimental protocol. This makes more straightforward the comparison between the two conditions of the changes in CSS excitability, which occur around the onset of the oculomotor response.

Under these quite demanding cognitive conditions for the oculomotor control, MEPs in relaxed muscles of the upper-limb exhibit an amplitude modulation, in the absence of any overt hand movement. In particular, data show a facilitation of CSS excitability of some distal upper-limb muscles, peaking approximately at 120 ms before the eyes begin to move, which comes back to baseline levels in correspondence of the saccade onset. Furthermore, a long-lasting, generalized decay in CSS excitability develops following the eye response. Interestingly, this effect is significantly deeper after prosaccades than after antisaccades. Finally, a quite large modulation of MEP amplitude, depending on the direction of the saccadic response, is observed in a narrow time window only in the FDI muscle, peaking at about 150 ms before saccade onset. This excitability modulation is independent of the type of the ocular response (pro-/anti-saccade) and appears to be tied up to the timing of the saccade onset, rather than being time-related to the occurrence of the visual cue.

### Direction-Specific CSS Excitability Modulation

Recent TMS studies have reported direction-specific changes of excitability in the upper-limb CSS in the absence of any manual response, following both reflexive saccades (Falciati et al., 2013) and volitional gaze shifts in a ‘go/no-go’ oculomotor task, in which the decision to make a foveating saccade was based on the color discrimination of a peripheral visual cue (Maioli and Falciati, 2015). Moreover, the CSS excitability changes were observed to occur after the execution of the eye response and to be tightly time-locked to the saccade onset. These data have been interpreted as supporting the viewpoint that, whenever the eyes move, a sub-threshold motor plan encoding an aiming movement of the hand toward the gaze target is also activated.

A modulation of CSS excitability in FDI muscles, as a function of the side the saccadic response is directed to, is also found in the ‘pro-/anti-saccade’ paradigm adopted in this study. Moreover, similarly to what was reported in our previous papers, changes in CSS excitability are congruent with the direction of the gaze shift. In fact, when the forearm is held in a fully pronated position, the FDI muscle is activated for a movement of the index finger toward the contralateral side and inhibited for a movement toward the ipsilateral side. Accordingly, CSS excitability of the right FDI muscle becomes significantly higher when the color of the visual cue indicates that a leftward saccade has to be made with respect to a rightward eye response, independently of whether a prosaccade or antisaccade has to be performed.

Present results are in agreement with our previous studies regarding the finding that the direction-specific modulation of MEP amplitude in FDI muscle is time-locked with the beginning of the eye response. However, they present also a remarkable discrepancy, inasmuch as the modulation in CSS excitability does not follow the eye movement, but it occurs before the saccade onset. This fact is at odd with the interpretation that this excitability modulation represents a covert motor plan for an aiming hand movement. In fact, many eye-hand coordination studies have repeatedly reported that hand movement starts between 70 and 90 ms after the initiation of the saccade and the hand takes up to 500 ms to arrive at the target (Carnahan and Marteniuk, 1994; Lünenburger et al., 2000; Sailer et al., 2000; Dean et al., 2011). By contrast, the direction-specific excitability modulation found in this study reaches its peak at 150 ms before the saccade onset and it is completely terminated before the eye begins to move. It can then be construed that the described direction-specific modulation of CSS excitability in the present study may be more linked to sensory/perceptual processes rather than to the execution of an upper-limb motor program.

This finding is somewhat unexpected, since, at a first glance, the execution of a prosaccade within the present experimental protocol does not seem to differ substantially from a saccade performed toward the peripheral cue in the ‘go/no-go’ paradigm (Maioli and Falciati, 2015). After all, in both double-choice tasks, participants make a decision of whether or not shifting their gaze toward a visual cue, depending on its color discrimination. This study was designed to verify whether a covert motor program for the hand is issued in a mandatory way, whenever an eye movement is executed. In the antisaccade paradigm a volitional saccade is made to the opposite direction of a visual cue, determining a spatial incongruence between sensory stimulus and eye motor response. Therefore, it constitutes an unusual behavioral condition in which a covert motor program for the hand could possibly be inhibited or, alternatively, generated with different spatial properties from those of the gaze shift. Actually, our data show that the modulation of MEP amplitude in FDI muscle is determined only by the saccade direction, independently of the type of the performed eye movement (pro-/anti-saccade). However, its occurrence before the eye movement execution makes it unlikely that this direction-specific modulation in MEP amplitude represents the activation of a hand motor program aiming at the same target of the ocular response. Rather, we hypothesize that it may constitute an activation of the motor system controlling the FDI muscle that reflects the outcome of the decision making process about the direction of the gaze shift to be performed.

An alternative explanation of the observed direction-specific modulation of CSS excitability could be inferred from the similarities existing between our findings and the increase in upper-limb CSS excitability described in simple manual reaction-time tasks (Rossini et al., 1988; Starr et al., 1988; Pascual-Leone et al., 1992; Chen et al., 1998; Leocani et al., 2000; Burle et al., 2002). In fact, the changes in CSS excitability preceding a voluntary arm motion are time-locked with movement onset and have been consistently reported to involve the agonist muscles in a directional manner. It has been suggested that this higher level of neural activation is related to the process of motor preparation and initiation of distal movements. In our experimental task, an actual hand movement is not required and, therefore, must be forcedly restrained. Therefore, a conceivable preparatory increase of motor excitability of the agonist muscles would be expected to be followed by an active inhibition of the motor program. Accordingly, our data demonstrate that a deep depression of the CSS excitability immediately follows the oculomotor response in all recorded muscles. However, if we accept this interpretation, it is hard to explain why in the similar ‘go/no-go’ paradigm (Maioli and Falciati, 2015) there is no trace of such a preparatory activity in the FDI muscle, preceding the execution of the eye movement. Finally, the hypotheses of a preparatory motor activation and of a reflection in the FDI motor map of the decision making process about which direction to direct the oculomotor response are not mutually exclusive.

In any case, our results clearly demonstrate that the behavioral context in which we redirect gaze in the visual scene heavily affects the manner in which eye and upper-limb motor systems are implicitly coupled.

### Pre-saccadic Motor Facilitation

Data show an increase in CSS excitability preceding the execution of the eye response in ADM and ECR muscles. The absence of a concomitant increase of MEP amplitude in the FDI muscle makes it unlikely that these excitability changes are just due to an unspecific activation of the arm representation in the primary motor cortex, consequent to the abrupt capture of visuo-spatial attention by a salient stimulus. Furthermore, the observed increase in CSS excitability appears to be synchronized with the execution of the eye movement, since it occurs in a definite time window starting about 200 ms before the ocular response (that is, at a variable latency following the visual cue) and ending in correspondence of the saccade onset, where it turns into a long lasting inhibition. Interestingly, there is a remarkable similarity between this finding and the results from the ‘go/no-go’ task (Maioli and Falciati, 2015), in which a peak of activation in motor excitability was found in all recorded muscles (including FDI) at approximately 150 ms before the ocular response. One could surmise that an increase of excitability in the hand cortical map could be finalized at enhancing the readiness of the upper-limb motor system to execute a possible object-oriented manual response. Furthermore, the fact that the transient increase in CSS excitability ends in correspondence of the execution of the eye response is compatible with the hypothesis that this sub-threshold motor facilitation is linked to the decision-making about which effector to move (i.e., whether or not the eye movement has to be accompanied also by an aiming movement of the hand). Finally, the differential involvement of the upper-limb muscles, between ‘go/no-go’ and ‘pro-/anti-saccade’ paradigms, may indicate that the facilitation in CSS excitability preceding saccade execution is not stereotyped, but once again it is determined by the particular behavioral set imposed by the cognitive demands of the task.

The described pre-saccadic motor facilitation in the present study could also be interpreted in the framework of the theory of a pragmatic representation of visual objects (Rizzolatti et al., 1994; Schneider and Deubel, 2002). According to this viewpoint, object attributes are represented as affordances, to the extent that they define possible specific motor patterns or schemas (Iberall and Arbib, 1990; Jeannerod, 1994). Thus, an activation of the upper-limb motor cortex could result from a suddenly appearing visual cue, as it could represent an achievable target for a customary motor response involving eye-hand coordination. Although this possibility cannot be fully excluded, the lack of directional specificity in the motor facilitation of ADM and ECR muscles argues against the hypothesis that the observed excitability changes represent a cortical pre-activation encoding a specific upper-limb motor program.

### Post-saccadic Depression of Motor Activity

Changes in CSS excitability after saccade execution also show clear differences between ‘go/no-go’ and ‘pro-/anti-saccade’ double-choice tasks.

In the ‘go/no-go’ task (Maioli and Falciati, 2015), CSS excitability quickly returns to baseline level after the ocular response in the ‘go’ trials. In addition, a side-dependent post-saccadic modulation of MEP amplitude is also present in FDI and ADM muscles, which is tightly time-locked to the onset the eye movement. In this study, by contrast, a pronounced long-lasting reduction in CSS excitability develops in all recorded muscles after the ocular response, with no direction-specific modulations in MEP amplitude. In other words, in place of a sub-threshold motor plan, encoding an aiming movement of the hand toward the gaze target, in the ‘pro-/anti-saccade’ task a depression of the motor activity is imposed to the upper-limb musculature. Interestingly, this reduction in CSS excitability is more marked after the execution of a prosaccade then after an antisaccade. On the basis of our data, it is hard to find a plausible explanation for this differential change of excitability. One can construe that the most common motor response in everyday life, which involves an eye-hand coordination toward a salient visual stimulus, requires a stronger depression of the upper-limb motor activity to be restrained, than during a very unnatural anti-saccade task.

Also in this case, the task-dependent differences in post-saccadic CSS excitability of the arm distal muscles provide compelling evidence for a crucial role of motor or behavioral set on oculo-manual response planning. In this respect, it can be argued that the main difference between ‘go/no-go’ and ‘pro-/anti-saccade’ protocols resides in the naturalness of the task. In fact, deciding whether or not to act toward a novel visual stimulus, as it occurs in the ‘go/no-go’ task, pertains to the repertoire of common behaviors in everyday life. Thus we can expect that, once a decision to act has been made, the selection of the motor response to activate falls on the prevailing motor pattern (or schema), involving a coordinated eye-hand motor plan. In other words, a co-activation of arm and eye muscles aiming toward a common spatial location would constitute a routine motor schema, which is triggered whenever a potential goal for motor action appears in the peripersonal space (Graziano et al., 1994; Rizzolatti et al., 1997). The viewpoint that arm movements are implicitly linked to gaze behavior is in accord with the large body of evidence demonstrating that, in natural conditions, gaze and arm movements are aimed at the same target and the accuracy of both systems is considerably enhanced if eye and hand move together (Vercher et al., 1994; Henriques et al., 1998; Lünenburger et al., 2000; Neggers and Bekkering, 2000, 2002; van Donkelaar and Staub, 2000; Medendorp and Crawford, 2002; Miall and Reckess, 2002; Horstmann and Hoffmann, 2005). Since a manual response is not required, the activation of the CSS is kept sub-threshold to prevent an upper-limb overt response.

In the ‘pro-/anti-saccade’ task, instead, subjects are asked to adopt a very unnatural behavior, i.e., deciding on the basis of the perceived cue color whether to make a saccade toward the visual stimulus or to its mirror image location on the horizontal meridian of an empty space. This is a condition in which the visual cue loses the attribute of being a possible goal to act on. Instead, the behavioral relevance of the cue resides just on its metric properties, which are essential for a correct saccade programming at the end of the decision-making process about the direction of the eye response. Along this line of reasoning, a coordinated eye-hand movement toward the visual cue becomes an aimless, competing motor program (or schema) that needs to be actively suppressed, since it would yield an undesirable interference within the behavioral context of the ‘pro-/anti-saccade’ task. Interestingly, a deep generalized inhibition of the CSS excitability takes place in all recorded muscles after the correct eye response is executed, unveiling an active suppression of the overall upper-limb motor activity. Such generalized post-saccadic decrease of CSS excitability below baseline does not occur during a more ‘natural’ behavioral context, as in the ‘go/no-go’ paradigm (Maioli and Falciati, 2015).

### Final Remarks

The concept of behavioral or motor set to describe the ability to reorganize behavior or motor responses conforming to the requirements of the task were proposed in the mid-70s (e.g., Buchwald et al., 1975) and, since then, it has been object of extensive investigation. It can be defined as “a state of brain activity which predisposes the subject to respond in one way when several alternatives are available” (Flowers and Robertson, 1985). Following this tenet, a motor response is not determined solely by the attributes of the sensory information, but it depends also on the internal state of the subject, according to the goal currently in operation. The volitional component of the task (instruction) plays a major role in shaping a motor plan or choosing a specific action among many competing possible motor responses. Several authors have proposed that basal ganglia are essential for motor pattern selection (Redgrave et al., 1999; Gurney et al., 2001; Mink, 2003; Bogacz and Gurney, 2007). A main finding of our study is that the set determined by the cognitive aspects of a specific sensorimotor task is reflected into the neural motor map of the limb musculature, as excitability changes of the CSS. This motor set representation may be related to cognitive processes (e.g., decision-making, perceptual analysis), to motor programming (e.g., aiming hand movements), as well as to a general facilitation or inhibition of muscle excitability, depending on the behavioral context of the task.

The finding that CSS motor representations can be modified by behavioral contingencies is in agreement with other data in the literature. It is well known that the simple observation of others’ actions (Fadiga et al., 1995, 2005) or the mental simulation of a movement (Izumi et al., 1995; Kasai et al., 1997; Hashimoto and Rothwell, 1999) induce changes in motor cortex excitability that are compatible with the observed or imagined action. However, changes in CSS excitability, recorded during imagery of a finger movement, are reduced when the imagined movement is incompatible with the subject’s hand posture (Vargas et al., 2004). Moreover, the motor representation in the CSS of an observed or imagined action is deeply affected by the upper-limb posture (Strafella and Paus, 2000; Maeda et al., 2002; Urgesi et al., 2006). Interestingly, posture-related changes in forearm CSS excitability have been also described during smooth pursuit eye movements, even if the task does not require any manual response (Maioli et al., 2007). Specifically, when subjects perform the eye pursuit by keeping the hand in a pronated posture (i.e., the normal posture during a manual tracking), changes in CSS excitability are compatible with a motor program for a hand movement aiming at the same target of the eyes. By contrast, when the eye pursuit is made with a supinated forearm, which is incongruent with the natural posture held during a manual tracking, no modulations of MEP amplitude are elicited in the limb musculature.

The observed changes in upper-limb CSS excitability provide further neurophysiological evidence for an implicit, tight coupling between the motor control systems of eyes and arm. However, the activation of a covert upper-limb motor plan, congruent with a manual movement aiming at the same target of the eyes, is not mandatory for every saccade execution, but depends on the motor task.

## Author Contributions

All authors listed have made a substantial, direct and intellectual contribution to the work, and approved it for publication.

## Conflict of Interest Statement

The authors declare that the research was conducted in the absence of any commercial or financial relationships that could be construed as a potential conflict of interest.
